# Light at the end of the tunnel of Corti

**DOI:** 10.1016/j.omtm.2023.05.006

**Published:** 2023-05-23

**Authors:** Kilian Hanlon, Hildegard Büning, Gwenaëlle S.G. Géléoc

**Affiliations:** 1 University College London, London, UK; 2 Transduction Consulting, London, UK; 3 Institute of Experimental Hematology, Hannover Medical School, Hanover, Germany; 4 REBIRTH - Research Center for Translational Regenerative Medicine, Hannover Medical School, Hanover, Germany; 5 Boston Children’s Hospital, Department of Otolaryngology, Boston, MA, USA

Optogenetics, first introduced in 2006 (Deisseroth et al.),^1^ takes advantage of photosensitive ion channels to manipulate cell activity with remarkable temporal and spatial resolution. The technique has been widely used to assess different cellular and biological functions (reviewed in Emiliani et al.^2^) and is now in clinical trials for vision restoration (NCT02556736, NCT03326336 at clinicaltrials.gov). While its application to the hearing field is more recent, progress has been made over the past decade to adapt this new tool for hearing restoration.^3^^,^^4^ In the current issue of *Molecular Therapy, Methods and Clinical Development*, Zerche et al. report the development of a new optogene that brings exciting prospects for the development of optical cochlear implants (oCIs) and their use in the clinic.

Currently, patients with moderate to profound sensorineural hearing loss who get little benefit from hearing aids may be candidates for a cochlear implant (CI). The CI is a neural prosthesis that translates sound signals collected through an external microphone into electrical impulses delivered to the auditory nerve via an electrode array embedded in the cochlea. This technology has revolutionized the field of hearing restoration. Many patients benefit from CIs and display improved speech perception in favorable acoustic environments. Furthermore, early implantation in children leads to greatly improved language acquisition skills. This technology, however, is not perfect. Clinical outcomes are highly variable, and CI patients struggle in noisy environments. Furthermore, while CI patients can perceive rhythm, they have more difficulties discriminating complex tones associated with music and melodies.

While CI technology has greatly improved since its inception, CIs are inherently limited by the spread of the electrical field from each electrode that contacts and activates a group of spiral ganglion neurons (SGNs). To circumvent this limitation oCIs are being developed that take advantage of laser stimulation to offer improved spatial and temporal resolution. Implementation of the strategy for hearing restoration involves the use of adeno-associated viral (AAV) vectors, injected into the ear, to express a photosensitive protein, typically a derivative of bacterial channelrhodopsin (ChR), in the SGNs. Expression of the photosensitive protein renders SGNs sensitive to optogenetic stimulation, which, thanks to novel, faster probes, enables faithful and improved frequency coding.^3^^,^^5^^,^^6^^,^^7^

Preclinical and clinical studies using ChR for optogenetic stimulation have taken advantage of compounds fused to fluorescent proteins (FPs). The work from Zerche et al. aims to develop and validate a new compound, devoid of a fluorescent tag, to limit possible adverse effects that may result from the introduction of a non-human sequence. Unfortunately, removal of FPs renders ChRs less excitable.^8^^,^^9^ The hypothesis is that the decrease in probe sensitivity is due to alterations in trafficking of the protein to the membrane. As such, Zerche et al. replaced EYFP in f-Chrimson with a set of C-terminal modifications coupled with a self-cleaving P2A sequence, allowing expression of two separate proteins. Specifically, Zerche et al. took advantage of plasma membrane trafficking sequences of the human potassium channel Kir2.1, which they fused to the C-terminal end of f-Chrimson. They assessed different versions of the trafficking domain and demonstrated that a minimal sequence provides optimal photocurrent density. This new fusion partner is derived from a human protein and is less likely to induce immune response. Furthermore, it is significantly smaller than previously used FPs (EYFP: 717 bp vs. TSKir2.1: 60 bp), which is favorable when using AAV vectors with limited cargo capacity (∼4.7kb).

Performance of the novel f-Chrimson-TSKir2.1 was assessed by injection of AAV2/9 vectors through the round window in postnatal P6-P7 mice and retroauricular insertion of a thin optical fiber into the scala tympani. Exposure to increasing light pulse intensity led to measurable optically evoked auditory brainstem responses (ABRs) at least up to 300 Hz with similar results for the new compound and f-Chrimson-EYFP.

While spatial resolution can be improved with oCI, temporal fidelity, which is essential for auditory function, remains a challenge. Although photosensitive channels with fast kinetics can allow better temporal coding for high-frequency stimuli, they are associated with decreased light sensitivity. In terms of temporal fidelity, the kinetics of channel closure is similar for f-Chrimson-TSKir2.1-P2A-Katsuhka and f-Chrimson-EYFP (4.9 ms). While still quite fast, f-Chrimson is slower than other optogenes.^5^ In terms of light sensitivity, the challenge is to accommodate fast optogenes while limiting phototoxicity. One way to compensate for low light sensitivity is to induce higher expression of the optogene.^10^ Indeed, the magnitude of the response observed in the Zerche et al. study was dependent upon the dose of AAV vectors. However, higher doses of the viral vectors led to SGN loss, indicating that over-expression of the optogene may be toxic. Additionally, Zerche et al. observed an apical/basal gradient of SGN transduction with lower percentages of SGNs transduced in the basal region. This is commonly seen in the inner ear, although new AAV vectors with improved efficacy are being developed to overcome this issue.^11^^,^^12^^,^^13^^,^^14^ Nevertheless, as highlighted in this report, AAV vector dosing will need to be carefully considered as this technology moves forward to the clinic.

In summary, the newly designed f-Chrimson-TSKir2.1 provides a new and improved optogenetic agent for oCIs that is smaller and potentially less immunogenic. This study provides an important step forward for the development and application of oCIs toward a novel clinical approach for the treatment of hearing loss. Outcomes from current clinical trials aimed at restoring vision using optogenetics may impact the application of this technology to the field of hearing restoration. Importantly, safety and tolerability measures will be crucial in validating the use of AAV vectors to transduce remaining SGNs in patients. AAV-based therapies have been approved for several conditions in recent years, including for RPE65-linked vision loss.^15^ Furthermore, the first clinical trials using AAV vectors targeting sensory cells of the inner ear have recently been approved (NCT05572073, NCT05821959 at clinicaltrials.gov). As such, a roadmap is now in place for the development of oCIs for hearing restoration in patients with sensorineural hearing loss.

## References

[1] Deisseroth K., Feng G., Majewska A.K., Miesenböck G., Ting A., Schnitzer M.J. (2006). Next-generation optical technologies for illuminating genetically targeted brain circuits. J. Neurosci..

[2] Emiliani V., Entcheva E., Hedrich R., Hegemann P., Konrad K.R., Lüscher C., Mahn M., Pan Z.-H., Sims R.R., Vierock J., Yizhar O. (2022). Optogenetics for light control of biological systems. Nat. Rev. Methods Primers.

[3] Dieter A., Keppeler D., Moser T. (2020). Towards the optical cochlear implant: optogenetic approaches for hearing restoration. EMBO Mol. Med..

[4] Hernandez V.H., Gehrt A., Reuter K., Jing Z., Jeschke M., Mendoza Schulz A., Hoch G., Bartels M., Vogt G., Garnham C.W. (2014). Optogenetic stimulation of the auditory pathway. J. Clin. Invest..

[5] Bali B., Lopez De La Morena D., Mittring A., Mager T., Rankovic V., Huet A.T., Moser T. (2021). Utility of red-light ultrafast optogenetic stimulation of the auditory pathway. EMBO Mol. Med..

[6] Dieter A., Duque-Afonso C.J., Rankovic V., Jeschke M., Moser T. (2019). Near physiological spectral selectivity of cochlear optogenetics. Nat. Commun..

[7] Mager T., Lopez De La Morena D., Senn V., Schlotte J., D Errico A., Feldbauer K., Wrobel C., Jung S., Bodensiek K., Rankovic V. (2018). High frequency neural spiking and auditory signaling by ultrafast red-shifted optogenetics. Nat. Commun..

[8] Gauvain G., Akolkar H., Chaffiol A., Arcizet F., Khoei M.A., Desrosiers M., Jaillard C., Caplette R., Marre O., Bertin S. (2021). Optogenetic therapy: high spatiotemporal resolution and pattern discrimination compatible with vision restoration in non-human primates. Commun. Biol..

[9] Klapoetke N.C., Murata Y., Kim S.S., Pulver S.R., Birdsey-Benson A., Cho Y.K., Morimoto T.K., Chuong A.S., Carpenter E.J., Tian Z. (2014). Independent optical excitation of distinct neural populations. Nat. Methods.

[10] Gupta N., Bansal H., Roy S. (2019). Theoretical optimization of high-frequency optogenetic spiking of red-shifted very fast-Chrimson-expressing neurons. Neurophotonics.

[11] Andres-Mateos E., Landegger L.D., Unzu C., Phillips J., Lin B.M., Dewyer N.A., Sanmiguel J., Nicolaou F., Valero M.D., Bourdeu K.I. (2022). Choice of vector and surgical approach enables efficient cochlear gene transfer in nonhuman primate. Nat. Commun..

[12] Ivanchenko M.V., Hanlon K.S., Devine M.K., Tenneson K., Emond F., Lafond J.F., Kenna M.A., Corey D.P., Maguire C.A. (2020). Preclinical testing of AAV9-PHP.B for transgene expression in the non-human primate cochlea. Hear. Res..

[13] Ivanchenko M.V., Hanlon K.S., Hathaway D.M., Klein A.J., Peters C.W., Li Y., Tamvakologos P.I., Nammour J., Maguire C.A., Corey D.P. (2021). A versatile capsid variant for transduction of mouse and primate inner ear. Mol. Ther. Methods Clin. Dev..

[14] Van Beelen E.S.A., Van Der Valk W.H., Verhagen T.O., de Groot J.C.M., Madison M.A., Shadmanfar W., Hensen E.F., Jansen J.C., Van Benthem P.P.G., Holt J.R., Locher H. (2022). Efficient viral transduction in fetal and adult human inner ear explants with AAV9-PHP.B vectors. Biomolecules.

[15] Maguire A.M., Bennett J., Aleman E.M., Leroy B.P., Aleman T.S. (2021). Clinical perspective: treating RPE65-associated retinal dystrophy. Mol. Ther..

